# Verbal overshadowing of face memory does occur in children too!

**DOI:** 10.3389/fpsyg.2013.00970

**Published:** 2013-12-24

**Authors:** Hedwige Dehon, Valentine Vanootighem, Serge Brédart

**Affiliations:** Cognitive Psychology Unit, Department of Psychology, University of LiègeLiège, Belgium

**Keywords:** verbal overshadowing, face processing, episodic memory, development, interference

## Abstract

Verbal descriptions of unfamiliar faces have been found to impair later identification of these faces in adults, a phenomenon known as the “verbal overshadowing effect” (VOE). Although determining whether children are good at describing unfamiliar individuals and whether these descriptions impair their recognition performance is critical to gaining a better understanding children's eyewitness ability, only a couple of studies have examined this dual issue in children and these found no evidence of VOE. However, as there are some methodological criticisms of these studies, we decided to conduct two further experiments in 7–8, 10–11, and 13–14-year-old children and in adults using a more optimal method for the VOE to be observed. Evidence of the VOE on face identification was found in *both* children and adults. Moreover, neither the accuracy of descriptions, nor delay nor target presence in the lineup was found to be associated with identification accuracy. The theoretical and developmental implications of these findings are discussed.

## Introduction

Child witnesses may in many instances be less accurate than adults (e.g., Ceci and Bruck, 1993; Bruck and Ceci, 1999): while they are as likely as adults to correctly identify a target face in a “target-present” lineup, they are often more likely to falsely recognize a foil in a “target-absent” lineup compared with adults (Pozzulo and Lindsay, 1998). Nevertheless, one of the first things asked of witnesses is to provide a description of the culprit. This description will in turn lead to the search for the culprit and to creating the lineup in which he/she will be scrutinized. Hence, knowing whether children are as good as adults at describing non-familiar individuals and whether this ability has any implication for their later recognition performance represents an important issue to consider. However, to date, this issue has not been adequately investigated.

Regarding the influence of description on identification in adults, research has shown that describing an unfamiliar face may significantly impair the ability to recognize this face in a subsequent lineup identification task, a phenomenon known as the “verbal overshadowing effect” (VOE) (Schooler and Engstler-Schooler, 1990). For instance, in their seminal study, (Schooler and Engstler-Schooler, 1990) presented participants with a video of a bank robbery, after which participants had to describe, or not, the facial features of the bank robber from memory. Compared with controls, participants who provided a description were subsequently less likely to identify the target face from a lineup. These results were supported more recently by meta-analyses (Meissner and Brigham, 2001; Meissner et al., 2008) revealing a small, but reliable, negative effect of verbal overshadowing (but see (Brown and Lloyd-Jones, 2005) for some evidence of a facilitation effect of verbalization on face recognition). Nevertheless, although the negative effect of verbalization has been widely documented, the mechanisms underlying this effect are not completely understood. Indeed, to date, three separate accounts have been proposed (e.g., see Chin and Schooler, 2008 for a review). The “*content*” or “*recoding interference*” account (Schooler and Engstler-Schooler, 1990; Meissner et al., 2001) proposes that verbal overshadowing arises because participants generate and later rely upon an inadequate verbal description of the content of their original non-verbal memory of the face. The “*transfer-inappropriate processing shift*” account (e.g., Schooler et al., 1997; Schooler, 2002) proposes that verbalization encourages the use of featural processing (instead of configural processing), which is less suitable for successful face recognition (e.g., Valentine, 1988; Tanaka and Farah, 1993). Finally, the “*criterion shift*” account (e.g., Clare and Lewandowsky, 2004) proposes that verbalization induces a conservative bias in people's responses, making participants less likely to make a positive identification from a lineup. Evidence exists for each account offered as an explanation for the VOE, although none is currently able to fit all the data reported in the literature (Chin and Schooler, 2008).

With respect to face processing and verbal overshadowing (VO) in children, there are good reasons to expect a VOE on face identification in children. Indeed, although it has long been suggested that children process faces in a more analytic/featural manner and are more highly influenced by paraphernalia (e.g., Carey and Diamond, 1977) compared with adults, it has been shown that configural coding is nevertheless reliably used early in development (e.g., Carey and Diamond, 1994; Freire and Lee, 2001; Gilchrist and McKone, 2003; Itier and Taylor, 2004). Consequently, children would also be susceptible to verbal overshadowing if their descriptions induce a shift in processing toward featural processing during the identification task, as suggested by the “*transfer-inappropriate processing shift*” account. Moreover, one might expect children's descriptions to be more general and include less accurate details than those of adults (Davies et al., 1989; Pozzulo and Warren, 2003). If the “*content*” account is correct, this may make children more likely to misidentify the target in a lineup based on a poorer verbal code. Although the question of whether, and to what extent, children and adults differ in the way they describe unfamiliar faces has been the topic of only a few studies, the majority of available data has shown that although children's descriptions are significantly less detailed than those of adults and adolescents (Davies et al., 1989; Pozzulo and Warren, 2003), they can be equally as accurate (Dent and Stephenson, 1979; Marin et al., 1979; Davies and Flin, 1988; Davies et al., 1989; Hutcheson et al., 1995; Dekle et al., 1996). Consequently, according to the “*content*” account, children would also be susceptible to verbal overshadowing if their more general descriptions act as a verbal misleading suggestion during the identification task. Alternatively, they might be less likely to be affected by the VOE if their less developed linguistic abilities make their descriptions of people shorter (Pozzulo and Warren, 2003). Thus, we might expect age and vocabulary performance to be associated with an increase in the number of reported descriptors which, in turn, would affect children's identification performance. Finally, we cannot exclude the possibility that, like adults, children might be less susceptible to identifying someone from a lineup if the verbalization makes their decision criteria stricter.

Nevertheless, very few studies have examined the influence of face description on subsequent identification performance in children. Memon and Rose (2002) presented 52 children (aged 8–9 years) with a staged event lasting about 8 min, during which an unfamiliar male entered the classroom, then walked into the classroom, presented the picture of a dog to the children, asked them whether they had seen his dog and left. After 24 h, the children were tested individually and were invited to complete a recognition test during which they were randomly assigned to one of four experimental conditions depending on whether they described the stranger or completed a comprehension test, on the one hand, and whether they were required to recognize his face from a “target-present” or a “target-absent” lineup, on the other hand. Before the recognition test, the children had been advised that the target face might or might not be presented in the lineup and that they could give a “don't know” answer. No effect of the condition (i.e., description vs. control) on lineup accuracy and no correlations between correct description and performance in the lineup were found. Consequently, the authors concluded that descriptions did not impact children's identification abilities. However, this study contains some methodological shortcomings, which render such a conclusion premature. Indeed, no group of adults was tested, so that we cannot rule out the possibility that the procedure used was not optimal for observing an overshadowing effect. In addition, the children may have been more interested in the picture of the dog rather than the stranger's face, and the duration of the time a child spent looking at the stranger's face might have been different for each child. In support of this argument, a closer inspection of the results seems to reveal that the children were overall better at recognizing the dog (i.e., 86.9%) than the stranger (i.e., 74.5%). In addition, the description and the control tasks were completed after a 24 h delay, which has sometimes been associated with the production of shorter descriptions than would not be long enough to elicit a VOE (Meissner and Brigham, 2001; Meissner et al., 2008). Finally, due to the number of factors examined (i.e., “description” vs. “control” condition, “target-present” vs. “target-absent” lineup) and the small sample size per condition used, Memon and Rose's study may have lacked statistical power. More recently, Karageorge and Zajac (2011; see also Zajac and Karageorge, 2009) have examined the effects of description on identification in two groups of children (aged 5–7 and 8–11 years). Because their study was not aimed at evaluating the occurrence of VOE, the authors did not directly manipulate the description condition (i.e., randomly assigning participants to either a “description” or “no description” condition). But, interestingly, they found no influence of description content on accurate identification in children that spontaneously described the target face compared to children that did not spontaneously describe the target face. However, some limitations comparable to those of Memon and Rose's (2002) study (i.e., no immediate test condition, no adult control group, a complex short staged event with no control of the presentation duration and/or the motivation to process the target face) can be found in this study. This means that it is difficult to rule out the possibility that children's identification performance could actually suffer from verbal overshadowing in other situations/conditions. While these studies do not allow strong and unequivocal conclusions to be drawn, they are, nevertheless, the only references in the literature to date regarding the effect of description on children's identification performance. In conclusion, although of theoretical and forensic importance, whether children are as likely as adults to show a VOE, whatever in immediate or delayed test conditions, is an issue that remains unclear.

In the present study, two experiments were conducted comparing several groups of children (aged 7–8, 10–11, 13–14 years, respectively) and one group of adult participants with a method that did not include any potential stimulus that could distract the participants from processing the target face and that presented the target in close-up during the whole presentation. Moreover, in contrast to previous studies, we used large samples of participants of different ages. Finally, we examined the effect of verbal description in an immediate test condition. In addition, the predictions of the “*content*,” “*processing shift*,” and “*criterion*” accounts of the effects of age on verbal overshadowing were considered. More precisely, the quality of the descriptions provided by the participants was assessed, and the relationship between description quality and identification accuracy in children and in adults was examined. According to the “*content*” account, because describing a face is difficult, the content of the description could be quite unspecific and/or contain some errors. Thus, descriptions might act as a misleading suggestion to the participants and have a detrimental effect on subsequent accurate lineup performance. Moreover, the “*content*” account also predicts that a strong relationship would be observed between the quantity and quality of verbalizations and identification accuracy. In addition, one might expect vocabulary performance and age to be associated with correct identification through the number of descriptors. According to the “*processing shift*” account, the act of describing a face (whatever the description is detailed or not) induces a shift from configural to featural processing, which would negatively affect participants' identification performance whatever the age group. Finally, the “*criterion*” account proposes that verbalization induces a conservative bias in responding, making participants less likely to make a positive identification from a lineup. Hence, participants in the description condition would make more miss responses (and, consequently, fewer false identifications) than the participants in the control condition, and this would be the case in any age group.

## Experiment 1

In addition to the general objectives of this study, the specific aim of this first experiment was twofold. Firstly, it was assessed whether inserting a delay between the encoding of the event and the description could explain the lack of evidence of VOE in children in the previous studies. Secondly, the effects of delay were also examined in order to contrast the predictions from the “*content*” and the “*processing shift*” accounts. Indeed, according to the “*content*” account, inserting a delay prior to verbalization (“*postencoding”* delay) would increase the likelihood of later incorrect identification due to the use of an inappropriate verbal code based on a weakened memory trace of the face. Alternatively, we cannot exclude the possibility that the descriptions produced after such a delay are so poor that they cannot elicit any VOE. According to the “*processing shift*” account, the description may affect the processes used during the later identification task and would operate at any age, leading also to a decrease in participants' performance. However, Karageorge and Zajac (2011) explored the effects of delay on verbal descriptions and lineup performance in children and found that younger children provided fewer descriptors than older children after 2 weeks. But, in contrast to the studies completed with adults, they found that despite this delay effect on the number of descriptors recalled after 2 weeks, description quantity did not exert a negative effect on children's identification accuracy. Similarly, with a 24-h “*postencoding*” delay, Memon and Rose (2002) found no correlation between description quality and identification performance, nor did they find evidence of verbal overshadowing.

In contrast, the few studies that have examined the effect of “*postdescription*” delay (ranging from 3 min to 2 days; see Meissner and Brigham, 2001, for a meta-analysis), showed a VOE when the identification task was performed either immediately or shortly after the description (i.e., after 10 min). However, in studies that used a longer delay (of at least 24 min), differences between the description and the control conditions were not significant, or they even revealed a verbal facilitation (Finger and Pezdek, 1999). According to the “*content*” account, when a delay is inserted between the description and the identification task, the fading memory of the reported description is less likely to act as a strong misleading suggestion to participants. In this case, the VOE does not occur because the verbal memory trace is less accessible than the original visual memory trace (Finger and Pezdek, 1999). That is, when identification of the target face is required long after verbalization, participants are more likely to rely on the original visual memory trace than on the verbal memory trace created during the description task. Alternatively, according to the “*processing shift*” account, after a delay between description and identification, participants are susceptible to shifting back toward a more holistic processing orientation crucial for correct identification. Therefore, both accounts suggest a release of verbal overshadowing in this condition, but the “*content*” account suggests that quality and/or quantity of description would be associated with identification performance. Provided that children also process faces in a configural manner, we would expect them to be able to shift back toward such processing after a 24-h delay, in the same way as adults. However, to our knowledge, no study has examined this specific issue in children.

## Method

### Participants

A total of 509 participants took part in the experiment. The sample included participants aged 7–8 years (*N* = 126, *M* age = 7.52, *SD* = 0.52, 65 girls), 10–11 years (*N* = 126, *M* age = 10.47, *SD* = 0.52, 63 girls,), 13–14 years (*N* = 126, *M* age = 13.48, *SD* = 0.5, 68 girls), and 18–30 years (*N* = 131, *M* age = 23.72, *SD* = 3.52, 69 females). All participants were native French speakers, with normal or corrected to normal vision.

#### Procedure and materials

***(1)Learning phase***. Participants were tested individually and were presented with a 2-min video-clip. They were instructed to pay attention to the video-clip and advised that they would be asked questions about what they had seen. The videotape depicted a 25-year-old Caucasian male (the face and shoulders visible; he was wearing a black t-shirt). The man's face had no visible distinctive sign such as a beard or other facial hair, glasses, or scars. During the 2 min, the individual did not speak, maintained a neutral facial expression and performed different neutral actions (e.g., knocking on the screen, moving forward and backward from the screen).

***(2)Description vs. Control task***. Participants were randomly assigned either to the description condition (the participants were instructed to spend 5 min verbally describing the previously seen target face from memory: “*Please describe in as much detail as possible the face that was presented to you on the videotape. Try to describe the person in sufficient detail so that someone else could identify him on the basis of your description*.”) or to the control condition (“*Please, try to give as many names as possible of four-legged animals*”). In addition, participants were also randomly assigned either to a “*no delay*,” a “24-h *postencoding*” delay or a “24-h ‘*postdescription/fluency*’ delay” condition in order to determine the influence of delay on the VOE. In the “*no delay*” condition, participants were presented with the 2-min video-clip, and then performed the description or fluency task and the identification task one after the other. In the “24-h ‘*postencoding*’ delay,” the description/fluency task and the identification task were performed a day after the video-clip presentation. Finally, in the “24-h ‘*postdescription*’ delay” condition, participants performed the identification task 1 day after they had been presented with the video-clip and had carried out the description/fluency task.

***(3)Identification task***. All the participants were instructed that they would be presented with a sequential lineup in which six faces would be shown successively. A “target-present” lineup was always used and faces were counterbalanced so that the target face and the foils appeared equally in each of the six possible ranks. The participants' task was to indicate, for each face, whether or not it was the target previously seen in the video. Participants were advised that the target person might or might not be in the lineup and that they could answer that the target was absent from the lineup (“not present” option). The test face stimuli were color head-and-shoulder photographs of Caucasian men that were unfamiliar to the participants (all were wearing the same black t-shirt and had no visible distinctive signs). Note that these stimuli had been extensively piloted with groups of independent adult participants. That is, the face stimuli were chosen to match a general description (see the “description” point in the results section for the creation of the description) of the target (approximately the same age, hair and eye color, face shape), with equivalent similarity ratings (ranging from 4.90 to 5.65 on a 7-point scale; “1” = not similar at all and “7” = very similar) and distinctiveness ratings (ranging from 2.10 to 3.05 on a 7-point scale; “1” = not distinctive at all and “7” = very distinctive) as the target face. Moreover, in order to be sure that the lineup was not biased toward the target face (see e.g., Malpass et al., 2009) lineup fairness was evaluated within two separate tasks (*N* = 20 in each task). In the first task, we provided a description of the target to the participants before they were presented with the “target-present” lineup and they were then asked to pick the person who fitted best with the previous description (i.e., a “forced-choice” condition). A chi square test indicated that the target face was not selected more often than the foils, χ^2^_(6)_ = 3.5, *p* = 0.74. In the second task, we simply informed another group of participants that a “person was suspected of having committed a crime” (i.e., without providing the description) before they were presented with the same lineup and they were then asked to pick the person they believed was the supposed culprit (i.e., a “forced choice” condition). Again, there were no significant differences in the frequency with which the different pictures were chosen, χ^2^_(6)_ = 6.98, *p* = 0.32.

***(4) Verbal abilities***. Finally, all the participants in the present experiment were administered the vocabulary subtest from the Wechsler Intelligence Scales (WISC IV and WAIS III; Wechsler, 1997, 2003). Raw scores were calculated for each participant.

## Results

An alpha level of 0.05 was set for all statistical tests, unless otherwise specified.

### Vocabulary performance

The raw score attained in the vocabulary subtest of the WISC III (for children) or WAIS IV (for adults) was used as an estimate of a participant's vocabulary skills. These scores were submitted to a 4 (Age) × 2 (Condition) × 3 (Delay) factorial ANOVA. The ANOVA revealed an effect of Age, *F*_(3, 485)_ = 221.28, *p* < 0.001. *Post-hoc* Tukey HSD tests revealed that vocabulary performance increased with age except that no significant difference was observed between 13–14-year-olds and adults. However, there was no main effect of condition, *F*_(1, 485)_ = 0.24, *p* = 0.63, delay, *F*_(2, 485)_ = 0.98, *p* = 0.38, or interaction (all *p*s > 0.10).

### Performance in the lineup

**Figure 1:**
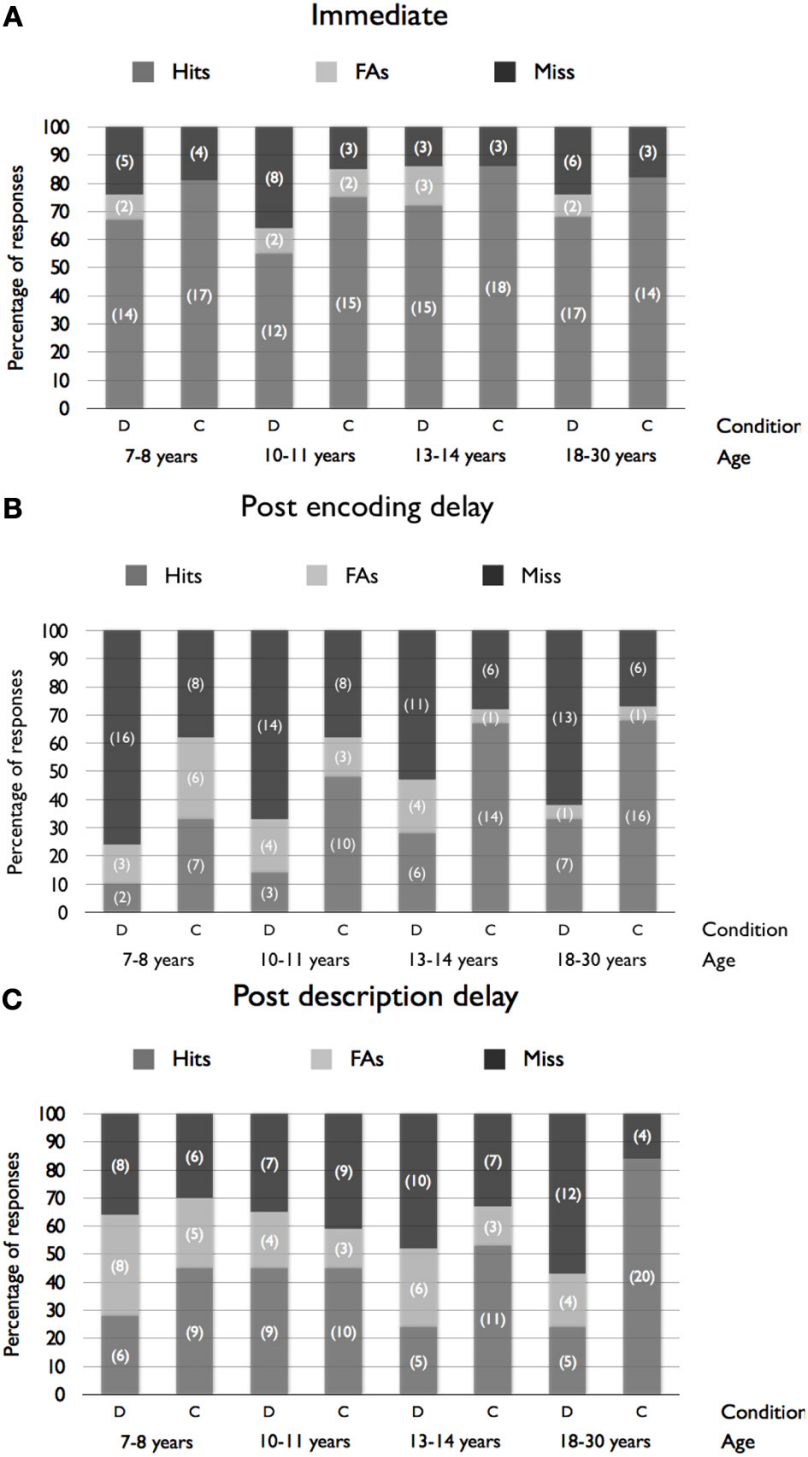
**Percentages of hits, false alarms (FAs) and miss responses as a function of Age and Condition for the immediate (A), postencoding delay (B) and postdescription delay (C) conditions (frequencies are presented in brackets)**.

The frequency of hits (i.e., correct identification), false alarms (i.e., identifying one of the five distractor faces) or misses (i.e., choosing the “not present” option) is presented in Figure 1 as a function of Age, Condition and Delay. Because of the small sample size in some cells, we were not able to examine the frequency of false alarm or miss responses separately. Hence, data were collapsed into binary responses (correct vs. ncorrect) to assess the effects of Age, Condition and Delay on lineup accuracy. The logit loglinear analysis revealed a main effect of Condition, χ^2^_(1)_ = 33.96, *p* < 0.001: participants in the description condition were less likely to correctly identify the target (*M* = 39%, *SD* = 21.7%) compared to the control group (*M* = 63.9%, *SD* = 18.3%). The analysis indicated a main effect of Age, χ^2^_(3)_ = 9.24, *p* = 0.03. *Post-hoc* analyses showed that younger children (aged 7–8 years; *M* = 44%, *SD* = 26.2%) made fewer correct identifications than adult participants (*M* = 59.8%, *SD* = 25.3%), while no significant differences were observed between the other groups. A main effect of delay, χ^2^_(2)_ = 45.90, *p* < 0.001, was also revealed. *Post-hoc* tests indicated that participants in the “*no delay*” condition made more correct identifications (*M* = 73.2%, *SD* = 10%), compared to participants in the two conditions including delay [*M*_(postencoding)_ = 37.6%, *SD* = 21.9%; *M*_(postdescription)_ = 43.5%, *SD* = 19.8%], while the difference between the two delay conditions was not significant. No interaction was revealed (all *p*s > 0.10). The absence of Age × Condition interaction, suggesting that the negative effect of description did not differ significantly across the four age groups, is of particular interest.

### Description

The following procedure was used in order to determine the accuracy of descriptions given by participants after viewing a target face. First, while viewing the target face, 8 independent scorers were asked to provide a detailed description of the target by using a checklist including 10 internal (e.g., eye color) and 5 external (e.g., hair color) features of the face, as well as 5 subjective personality characteristics (e.g., serious). An internal or external feature was considered to be correct if it matched the description of at least 85% of the scorers (e.g., *brown hair* or *brown eyes*). However, because we believed that more subjectivity might operate for the estimation of personality characteristics than for faces, these evaluations were considered as a separate category (i.e., “subjective descriptors”; see Finger and Pezdek, 1999; Meissner et al., 2001; Finger, 2002; Meissner, 2002). During Experiment 1, participants' descriptions were recorded and later processed by two independent coders, who classified the descriptors included in each face description into three categories: correct, incorrect or subjective. A Cohen's kappa test was performed to examine the general agreement between the two independent coders in the classification of the descriptors and the results indicated an overall agreement of 94.6%.

In order to compare the quality of description across age groups, a 4 (Age) × 3 (Delay) × 2 (Descriptor Type: Correct, Incorrect and Subjective) with repeated measures on the last factor was run on the number of descriptors reported (see Table 1). The analysis revealed a main effect of the types of descriptor, *F*_(2, 490)_ = 947.25, *p* < 0.0001. Planned comparisons indicated that participants were far more likely to produce correct descriptors (*M* = 3.14, *SD* = 1.33) than incorrect descriptors (*M* = 0.69, *SD* = 0.72) and more incorrect than subjective (*M* = 0.24, *SD* = 0.447) descriptors.

**Table 1:** **Mean number of reported descriptors in Experiment 2 as a function of Age and Delay (standard deviations are presented in brackets)**.

	**7–8**	**10–11**	**13–14**	**Adults**
**IMMEDIATE**				
Correct	2.72 (0.77)	3.83 (0.78)	4.48 (1.12)	5.09 (1.31)
	*r*_*pb*_ = 0.27, *p* = 0.22	*r*_*pb*_ = 0.03, *p* = 0.89	*r*_*pb*_ = 0.27, *p* =0.23	*r*_*pb*_ = 0.05, *p* = 0.82
Incorrect	0.45 (0.51)	0.43 (0.51)	0.33 (0.58)	0.39 (0.58)
	*r*_*pb*_ = −0.16, *p* = 0.48	*r*_*pb*_ = −0.11, *p* = 0.6	*r*_*pb*_ = 0.00, *p* = 1	*r*_*pb*_ = −0.14, *p* = 0.53
Subjective	0.23 (0.43)	0.35 (0.57)	0.33 (0.66)	0.39 (0.65)
	*r*_*pb*_ = 0.14, *p* = 0.54	*r*_*pb*_ = 0.07, *p* = 0.73	*r*_*pb*_ = −0.33, *p* = 0.15	*r*_*pb*_ = 0.15, *p* = 0.49
Total	3.41 (0.91)	4.61 (0.66)	5.14 (0.85)	5.87 (1.04)
	*r*_*pb*_ = 0.20, *p* =.36	*r*_*pb*_ = 0.01, *p* = 0.96	*r*_*pb*_ = 0.11, *p* = 0.64	*r*_*pb*_ = 0.17, *p* = 0.43
**POST ENCODING DELAY**				
Correct	1.86 (0.85)	2.60 (0.68)	2.7 (0.80)	3.59 (1.18)
	*r*_*pb*_ = 0.23, *p* = 0.32	*r*_*pb*_ = 0.00, *p* = 1	*r*_*pb*_ = −0.12, *p* = 0.61	*r*_*pb*_ = 0.23, *p* = 0.17
Incorrect	0.71 (0.56)	0.60 (0.68)	0.85 (0.74)	1.00 (0.82)
	*r*_*pb*_ = 0.25, *p* = 0.27	*r*_*pb*_ = 0.17, *p* = 0.46	*r*_*pb*_ = 0.15, *p* = 0.52	*r*_*pb*_ = 0.46, *p* = 0.03
Subjective	0.14 (0.36)	0.20 (0.41)	0.30 (0.47)	0.23 (0.43)
	*r*_*pb*_ = −0.19, *p* = 0.39	*r*_*pb*_ = −0.29, *p* = 0.22	*r*_*pb*_ = −0.02, *p* = 0.92	*r*_*pb*_ = −0.12, *p* = 0.47
Total	2.71 (0.85)	3.40 (0.50)	3.85 (0.67)	4.82 (0.85)
	*r*_*pb*_ = 0.31, *p* = 0.16	*r*_*pb*_ = 0.00, *p* = 1	*r*_*pb*_ = 0.008, *p* = 0.97	*r*_*pb*_ = −0.04, *p* = 0.86
**POST DESCRIPTION DELAY**				
Correct	1.86 (0.85)	2.10 (0.79)	3.14 (0.64)	3.45 (1.01)
	*r*_*pb*_ = 0.23, *p* = 0.30	*r*_*pb*_ = −42, *p* = 0.06	*r*_*pb*_ = −0.01, *p* = 0.95	*r*_*pb*_ = 0.03, *p* = 0.90
Incorrect	0.71 (0.72)	0.90 (0.72)	1.00 (0.87)	1.00 (0.87)
	*r*_*pb*_ = −0.04, *p* = 0.85	*r*_*pb*_ = 0.47, *p* = 0.04	*r*_*pb*_ = 0.33, *p* = 0.13	*r*_*pb*_ = −0.12, *p* = 0.59
Subjective	0.09 (0.30)	0.15 (0.37)	0.18 (0.39)	0.23 (0.43)
	*r*_*pb*_ = 0.15, *p* = 0.50	*r*_*pb*_ = 0.23, *p* = 0.33	*r*_*pb*_ = −0.11, *p* = 0.62	*r*_*pb*_ = 0.30, *p* = 0.16
Total	2.67 (1.11)	3.15 (0.81)	4.32 (1.04)	4.68 (1.04)
	*r*_*pb*_ = 0.19, *p* = 0.39	*r*_*pb*_ = 0.10, *p* = 0.66	*r*_*pb*_ = 0.23, *p* = 0.31	*r*_*pb*_ = −0.01, *p* = 0.97

*Point bi-serial correlations between descriptors and identification accuracy (and their respective p-values) are also presented in italics. A Bonferroni correction was applied so that a correlation was significant if p < 0.003*.

In addition, overall, the number of descriptors reported increased with age, *F*_(3, 245)_ = 73.97, *p* < 0.0001. *Post-hoc* HSD tests showed that the number of descriptors significantly increased at each age [*M*_(7−−8 years_) = 2.94, *SD* = 1.01; *M*_(10−11 years_) = 3.76, *SD* = 0.93; *M*_(13−−14 years_) = 4.44, *SD* = 1.01; *M*_(adults)_ = 5.13, *SD* = 1.11]. However, a significant Age × Descriptor Type interaction was significant, *F*_(6, 490)_ = 18.74, *p* < 0.0001. HSD tests revealed that although the number of correct descriptors increased with age, the number of incorrect and subjective descriptors was very similar across age (see Table 1). There was also a significant main effect of Delay, *F*_(2, 245)_ = 41.54, *p* < 0.0001. HSD tests showed that participants provided more descriptors in the “*no delay*” condition (*M* = 4.76, *SD* = 1.25) than participants in the “*postencoding* delay” (*M* = 3.71, *SD* = 1.06) and participants in the “*postdescription* delay” condition (*M* = 3.73, *SD* = 1.29). There was no significant difference between participants submitted to the last two delay conditions Finally, the analysis revealed a significant Delay x Descriptor Type interaction *F*_(4, 490)_ = 38.66, *p* < 0.0001. HSD tests showed that participants in the “*no delay*” condition provided more correct and fewer incorrect descriptors than participants in the other two delay conditions, while the number of subjective details did not differ significantly across the three delay conditions.

Point bi-serial correlations were used to examine correlations between identification accuracy and description quality. No significant correlation between description quantity (as measured as the total number of descriptors provided) and face identification was found in any age group (see Table 1). In addition, no significant correlation between description accuracy (whether measured as either the number of correct or incorrect descriptors) and face identification was found in any age group or any delay condition.

Finally, performance on the vocabulary task (raw scores) was not significantly correlated with the number of descriptors recalled or with the accuracy of the description in each age group of any delay condition (see Table 2).

**Table 2:** **Correlations between performance on the vocabulary task and description quantity (A), description quality (B and C) and identification performance (D)**.

	**7–8**	**10–11**	**13–14**	**Adults**
**A**	**Vocabulary and total number of descriptors (Pearson's correlations)**			
No delay	*r* = 0.04	*r* = −0.33	*r* = −0.27	*r* = −0.01
Post encoding delay	*r* = −0.20	*r* = 0.42	*r* = −0.15	*r* = 0.13
Post description delay	*r* = 0.16	*r* = −0.22	*r* = −0.15	*r* = 0.10
**B**	**Vocabulary and number of correct descriptors (Pearson's correlations)**			
No delay	*r* = 0.21	*r* = −0.19	*r* = −0.01	*r* = 0.12
Post encoding delay	*r* = −0.21	*r* = 0.08	*r* = −0.22	*r* = −0.20
Post description delay	*r* = 0.14	*r* =0.12	*r* = −0.04	*r* = 0.11
**C**	**Vocabulary and number of incorrect descriptors (Pearson's correlations)**			
No delay	*r* = −0.02	*r* = −0.17	*r* = −0.57	*r* = 0.14
Post encoding delay	*r* = −0.05	*r* = 0.11	*r* = 0.05	*r* = 0.19
Post description delay	*r* = 0.27	*r* = −0.26	*r* = 0.00	*r* = −0.08
**D**	**Vocabulary and identification performance (Point bi-serial correlations)**			
No delay	*r*_*pb*_ = −0.14	*r*_*pb*_ = 0.19	*r*_*pb*_ = 0.09	*r*_*pb*_ = 0.14
Post encoding delay	*r*_*pb*_ = −0.22	*r*_*pb*_ = −0.24	*r*_*pb*_ = 0.01	*r*_*pb*_ = −0.44
Post description delay	*r*_*pb*_ = −0.34	*r*_*pb*_ = 0.23	*r*_*pb*_ = 0.22	*r*_*pb*_ = −0.09

*After a Bonferroni correction was applied, a value of p < 0.004 was considered significant*.

## Discussion

The present experiment showed that describing a target face had a detrimental effect on the subsequent accurate lineup performance in both children and adults. We also found that age increased the number of accurate descriptors produced but not the number of incorrect descriptors. This suggests, in agreement with previous literature, that children produce less detailed, but not less accurate, descriptions than do adults (e.g., Davies and Flin, 1988; Davies et al., 1989; Dekle et al., 1996; Pozzulo and Warren, 2003). The presence of the VOE in immediate test conditions in all the age groups may support both the “*content*” and the “*processing shift*” accounts. However, the absence of correlation between descriptor accuracy, vocabulary performance and correct identification is more consistent with the “*processing shift*” account. Indeed, it was not the quantity or even the quality of the descriptors produced that impacted identification accuracy but, rather, the mere fact of verbally describing the face (and presumably shifting to featural processing).

In addition, we found that the negative effect of verbalization remained when a 24-h delay was inserted after face encoding (i.e., before the verbalization) in both children and adults. At first glance, such a result, albeit contrasting with previous studies examining the effects of description on children's identification performance (Memon and Rose, 2002; Karageorge and Zajac, 2011), may also support both the “*content*” and the “*processing shift*” accounts. Indeed, according to the “*content*” account, inserting a delay prior to verbalization would decrease description accuracy due to an impairment of the original memory trace, thereby increasing the likelihood of later incorrect identification. However, a closer inspection of the data does not fit this explanation of the occurrence of the VOE. Indeed, as in the immediate test condition, in the “*postencoding* delay” condition, no relationship was found between identification performance and the quantity or the quality of the descriptions. Nevertheless, data from the “*postencoding* delay” condition remain consistent with the “*processing shift*” account.

Finally, we also found a VOE in children and adults in the “*postdescription*” condition, and this result supports neither the “*content*” nor the “*processing shift*” accounts. Indeed, according to the “*content*” account, when a delay is inserted between the description and the identification task, the fading memory of the reported description would be less likely to act as a strong misleading suggestion to our participants and, consequently, to have a detrimental effect on subsequent lineup performance. At variance with such a prediction, no release of verbal overshadowing was found: there was no difference in identification performance between the “*postencoding* delay” and the “*postdescription* delay” conditions. In addition, also in the “*postdescription* delay” condition, there was no relationship between the content of descriptions and identification performance. Similarly, the “*processing shift*” account predicts a release of the VOE in the “*postdescription* delay” condition because participants would be susceptible to shifting back toward a more holistic processing orientation when a long delay is inserted after verbalization, but such a release did not occur here. Finally, in this experiment, we were not able to examine the relevance of the “*criterion*” account to explain our results. Indeed, conditions were not optimal for taking into account the kind of errors (misses or false alarms) committed by the participants in the lineup identification task. This point was considered further in Experiment 2 with the use of a “target-absent” lineup.

## Experiment 2

The use of a “target-absent” lineup is interesting in assessing the prediction of the “*criterion shift*” account more directly. That is, if the “*criterion shift*” account is correct, we might predict that verbalization would decrease the participants' correct identification in the “target-present” condition, while verbalization would increase correct rejection in the “target-absent” condition. Indeed, if the recognition criterion become stricter after verbalization, adult participants would be reluctant to choose someone in the lineup and, hence, make more incorrect rejections (i.e., miss responses) and fewer false alarms when the target is present than in the control condition. However, they would make more correct rejections and fewer false alarms when the target is absent from the lineup than in the control condition. Examining the VOE with a “target-absent” lineup in adults, Clare and Lewandowsky (2004) found evidence of a VOE on a “target-present” lineup only when participants were provided with a “not present” option, while they found no such effect when “target-absent” lineups were used. Rather, when “target-absent” lineups were used, verbalization improved accurate performance, as the conservative bias led participants to made fewer false identifications. Sauerland et al. (2008, see also Meissner, 2002) have confirmed these results, while other researchers have been unable to replicate them (e.g., Fallshore and Schooler, 1995).

We might make the same predictions for children, although it could be expected from previous research that they would be more prone to select someone in the “target-absent” lineup condition than would adults. Indeed, a meta-analysis by Pozzulo and Lindsay (1998) indicated that whereas correct identification does not differ significantly in children, when comparing 5 year-olds and adults, children are less likely than adults to make a correct rejection and more likely to choose someone from a lineup in which the culprit is absent (e.g., Wells and Luus, 1990; Parker and Ryan, 1993; Zajac and Karageorge, 2009). More relevant to the current study, in their experiment examining the impact of description on face identification in children, Memon and Rose (2002) found that 8–9 year-olds were more accurate in “target-present” (74.5%) than in “target-absent” lineups (42%), in which 58% of children were unable to correctly reject the lineup.

In addition, because the failure to observe a correlation between the quality of description and the identification performance could be due to particularities of the specific groups used in Experiment 1, we examined again whether the quality of the descriptions was related to identification accuracy in children and adults. Overall, we predicted that the number of descriptors would increase with age. More specifically, following the “*content*” account, the quality of descriptions would be expected to be related to identification accuracy in both children and adults. Moreover, vocabulary scores would have correlated with the number of descriptors reported which, in turn, would affect identification accuracy in all the groups. However, if verbalization acts as a misleading suggestion, more false alarms and fewer miss responses would be expected to occur in both “target-present” and “target-absent” lineups compared with their respective control conditions. If the “*processing shift*” account is correct, the simple act of describing the target face would induce a more featural processing instead of a configural processing during the recognition task, leading to more miss responses than false alarms in both kinds of lineup. As, in Experiment 1, the delay was not found to explain the discrepancies between our results and those from previous studies examining the effect of verbal description on children's face recognition (i.e., Memon and Rose, 2002; Karageorge and Zajac, 2011), a “*no delay*” condition only was used in Experiment 2.

## Methods

### Participants

Four groups of 80 participants (40 females in each) were included: 7–8 year olds (*M* = 7.49, *SD* = 0.5); 10–11 year olds (*M* = 10.52, *SD* = 0.5); 13–14 year olds (*M* = 13.42, *SD* = 0.52) and adults (*M* = 23.59, *SD* = 2.88). All the 320 participants were native French speakers, with normal or corrected to normal vision.

### Procedure and materials

The design, materials and procedure were identical to those of Experiment 1 except that all the participants were tested in “*no delay*” conditions. In addition, half of the participants were presented with a “target-absent” lineup, while the other half was presented with a “target-present” lineup during the identification task.

## Results

### Vocabulary performance

The effects of Age (7–8, 10–11, 13–14 years and adults), Condition (description vs. control) and Target presence (present vs. absent) were examined on the raw scores on the vocabulary task to ensure that the participants were not significantly different in term of verbal ability. A factorial ANOVA revealed an effect of Age [*F*_(3, 304)_ = 114.24, *p* < 0.001]. *Post-hoc* HSD analyses showed that performance on the vocabulary task increased significantly with age, except that no significant difference was found between 13–14-year-old children and adults. Analyses also showed a main effect of Target presence, [*F*_(1, 304)_ = 112.54, *p* < 0.001]. *Post-hoc* analyses revealed that vocabulary performance was significantly lower in participants in the “target-present” lineup condition (*M* = 28.46, *SD* = 6.79), compared with participants in the “target-absent” lineup condition (*M* = 34.74, *SD* = 8.35). However, analyses revealed no effect of Condition, *F*_(1, 304)_ = 0.32, *p* = 0.57. No significant interaction between these factors was found.

### Performance in the lineup

**Figure 2:**
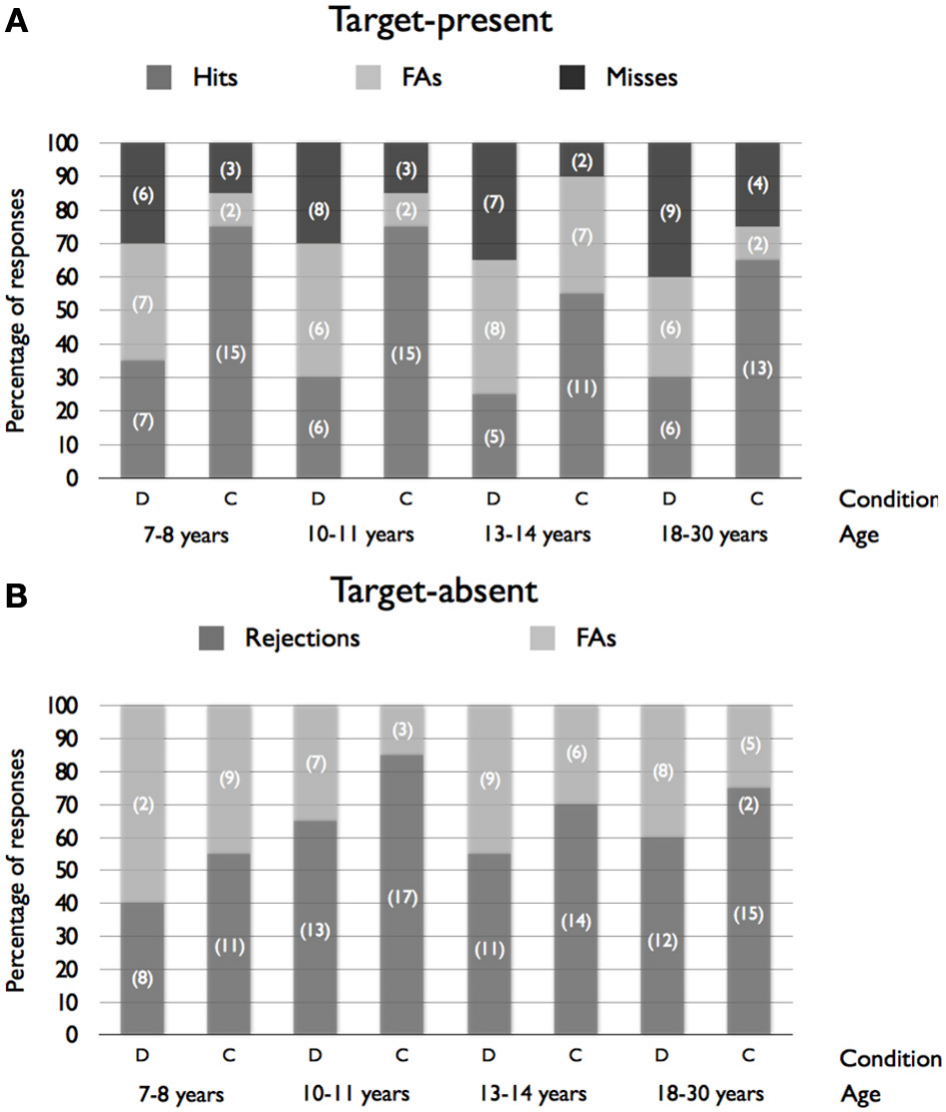
**Percentages of hits, false alarms (FAs), miss responses and rejections as a function of Age and Condition for target-present (A) and target-absent (B) conditions (frequencies are presented in brackets)**. Note that a correct response was represented by the identification of the target in the “target-present” lineup but choosing no one in the “target-absent” lineup.

The frequency of responses in both types of lineup is presented in Figure 2. Data were collapsed into binary responses (correct and incorrect performance) in order to examine the effects of Age (7–8, 10–11, 13–14 years and adults), Condition (description vs. control) and Target (present vs. absent) on lineup accuracy. Note that a correct response was represented by the identification of the target in the “target-present” lineup but choosing no one in the “target-absent” lineup, otherwise the response was considered as incorrect. Following the method of Memon and Rose (2002), performance in the “target-present” lineup and in the “target-absent” lineup was submitted to the same statistical analysis. A logit loglinear analysis made on these binary responses revealed a main effect of Condition, χ^2^_(1, *N* = 320)_ = 22.31, *p* < 0.001. Overall, participants who provided a description were subsequently less likely to perform correctly during the lineup identification (42.5%, *SD* = 15.3%) compared to the control group (69.4%, *SD* = 10.5%). Analysis also revealed a main effect of target presence on accurate lineup performance, showing that correct performance was better in the “target-absent” condition than in the “target-present” condition, χ^2^_(1)_ = 6.13, *p* < 0.01. In other words, it was easier to correctly reject a foil in the “target-absent” lineup than to correctly identify the target in the “target-present” lineup. However, no main effect of Age, χ^2^_(3)_ = 3.37, *p* = 0.34, no Age × Condition interaction, χ^2^_(3)_ = 0.95, *p* = 0.81, no Target presence × Condition interaction, χ^2^_(1)_ = 2.53, *p* = 0.11, and no Age × Target presence, χ^2^_(3)_ = 5.26, *p* = 0.15, were observed. Finally, the three-way Age × Condition × Target presence was not significant, χ^2^_(3)_ = 0.29, *p* = 0.96. As in Experiment 1, the absence of Age × Condition interaction, suggesting that the negative effect of description did not differ significantly across the four age groups, is of particular interest.

Because of the low frequency of miss and false alarm responses in the “target-present” lineup, we were not able to compare those two categories of response in each experiment separately. Hence, data from identical conditions (i.e., the “target-present” lineups with “*no delay*”) from the two experiments were collapsed in order to examine the types of error (misses vs. false alarms) made by participants in the description and control conditions in the 4 groups of participants. A logit loglinear analysis revealed a main effect of Condition, χ^2^_(1)_ = 5.21, *p* = 0.02. Overall, participants assigned to the control condition produced more false alarms (46.5%, *SD* = 10.8%) than participants who provided a description (30.1%, *SD* = 28.3%). However, no main effect of Age, χ^2^_(3)_ = 0.36, *p* = 0.95 and no significant Condition × Age interaction, χ^2^_(3)_ = 1.73, *p* = 0.63 were observed.

In addition, because the “*criterion shift*” account led us to predict that in the “target-absent” lineup, the rates of false alarms would be lower in the “description” condition than in the “control” condition, an analysis comparing these rates was performed separately in the “target-absent” condition. A logit loglinear analysis revealed a main effect of Condition, χ^2^_(1)_ = 4.84, *p* = 0.03. Analysis revealed that false alarms were more frequent in the “description” condition (47%) than in the “control” condition (28.7%). However, no main effect of Age, χ^2^_(3)_ = 7.05, *p* = 0.07 and no significant Condition × Age interaction, χ^2^_(3)_ = 0.29, *p* = 0.96 were observed.

### Description

The Cohen's kappa test performed to examine the general agreement between the two independent coders in the classification of the descriptors indicated an overall agreement of 91.05%. In order to compare the quality of description across development, a 4 (Age) × 2 (Target: present vs. absent) × 2 (Descriptor Type: Correct, Incorrect and Subjective) with repeated measures on the last factor was run on the number of descriptors reported (see Table 3). Overall, a main effect of Descriptor type was revealed, *F*_(2, 304)_ = 347.70, *p* < 0.0001. Planned comparisons indicated that participants were far more likely to produce correct descriptors (*M* = 2.96, *SD* = 1.33) than incorrect descriptors (*M* = 0.79, *SD* = 0.84). Participants also produced more incorrect than subjective descriptors (*M* = 0.26, *SD* = 0.49). The ANOVA also showed a main effect of Age, *F*_(3, 156)_ = 33.87, *p* < 0.0001. HSD Tukey tests indicated that the number of descriptors increased gradually with age, although no significant difference was found between 10 and 11 and 13 and 14-year-old children. Moreover, there was a significant Age × Descriptor Type interaction, *F*_(3, 312)_ = 12.26, *p* < 0.0001. HSD tests showed that the number of correct descriptors increased with age, although no significant difference was found between 10 and 11 and 13 and 14-year-old children. In addition, the number of incorrect descriptors and the number of subjective descriptors were very low in general and did not significantly change across age. Finally, no main effect of Target presence was observed, *F*_(1, 152)_ = 3.21, *p* = 0.075.

**Table 3:** **Mean number of reported descriptors in Experiment 2 as a function of Age and Target presence (standard deviations are presented in brackets)**.

	**7–8**	**10–11**	**13–14**	**Adults**
**TARGET-PRESENT**				
Correct	2.10 (0.91)	2.60 (0.94)	2.70 (1.03)	3.75 (1.12)
	*r*_*pb*_ = 0.01, *p* = 0.96	*r*_*pb*_ = −0.06, *p* = 0.78	*r*_*pb*_ = −0.24, *p* = 0.30	*r*_*pb*_ = −0.14, *p* = 0.56
Incorrect	0.60 (0.75)	1.00 (0.92)	0.75 (0.72)	0.75 (0.92)
	*r*_*pb*_ = 0.08, *p* = 0.73	*r*_*pb*_ = −0.34, *p* = 0.15	*r*_*pb*_ = 0.15, *p* = 0.54	*r*_*pb*_ = 0.51, *p* = 0.02
Subjective	0.25 (0.55)	0.20 (0.41)	0.30 (0.47)	0.35 (0.59)
	*r*_*pb*_ = −0.23, *p* = 0.32	*r*_*pb*_ = −0.05, *p* = 0.83	*r*_*pb*_ = 0.53, *p* = 0.01	*r*_*pb*_ = 0.26, *p* = 0.26
Total	2.95 (1.14)	3.80 (1.04)	3.75 (0.85)	4.85 (1.00)
	*r*_*pb*_ = −0.05, *p* = 0.84	*r*_*pb*_ = −0.43, *p* = 0.06	*r*_*pb*_ = 0.12, *p* = 0.60	*r*_*pb*_ = 0.41, *p* = 0.07
**TARGET-ABSENT**				
Correct	1.90 (0.91)	2.80 (0.83)	2.85 (1.27)	4.95 (1.96)
	*r*_*pb*_ = −0.14, *p* = 0.56	*r*_*pb*_ = −0.33, *p* = 0.16	*r*_*pb*_ = −0.18, *p* = 0.44	*r*_*pb*_ = 0.29, *p* = 0.20
Incorrect	0.60 (0.68)	0.55 (0.76)	1.05 (0.82)	1.05 (1.05)
	*r*_*pb*_ = 0.18, *p* = 0.44	*r*_*pb*_ = 0.19, *p* = 0.41	*r*_*pb*_ = −0.08, *p* = 0.75	*r*_*pb*_ = 0.24, *p* = 0.31
Subjective	0.20 (0.41)	0.25 (0.55)	0.25 (0.55)	0.25 (0.44)
	*r*_*pb*_ = −0.15, *p* = 0.52	*r*_*pb*_ = 0.19, *p* = 0.42	*r*_*pb*_ = 0.00, *p* = 1	*r*_*pb*_ = 0.00, *p* = 1
Total	2.70 (0.98)	3.60 (0.94)	4.15 (0.99)	6.25 (2.05)
	*r*_*pb*_ = −0.06, *p* = 0.79	*r*_*pb*_ = −0.02, *p* = 0.93	*r*_*pb*_ = −0.29, *p* = 0.20	*r*_*pb*_ = 0.38, *p* = 0.10

*Point bi-serial correlations between descriptors and identification accuracy (and their respective p-values) are also presented in italics. After a Bonferroni correction was applied, a value of p < 0.003 was considered significant*.

No significant correlation between description quantity (as measured as the number of descriptors provided) or quality (whether measured as the number of correct or incorrect descriptors) and face identification was found (see 3).

Finally, as shown in Table 4, vocabulary performance was associated neither with quantity of descriptors, nor quality of description nor identification performance in any age or lineup condition.

**Table 4:** **Correlations between performance on the vocabulary task and description quantity (A), description quality (B and C) and identification performance (D)**.

	**7–8**	**10–11**	**13–14**	**Adults**
**A**	**Vocabulary and total number of descriptors (Pearson's correlations)**			
Target-present	*r* = 0.09	*r* = 0.27	*r* = −0.11	*r* = −0.07
Target-absent	*r* = 0.44	*r* = −0.12	*r* =-0.27	*r* = 0.26
**B**	**Vocabulary and number of correct descriptors (Pearson's correlations)**			
Target-present	*r* = −0.23	*r* = 0.22	*r* = −0.45	*r* = 0.13
Target-absent	*r* = 0.33	*r* = −0.02	*r* = 0.01	*r* = 0.48
**C**	**Vocabulary and number of incorrect descriptors (Pearson's correlations)**			
Target-present	*r* = 0.49	*r* = 0.15	*r* = 0.41	*r* = −0.16
Target-absent	*r* = 0.19	*r* = −0.23	*r* = 0.06	*r* = −0.18
**D**	**Vocabulary and identification performance (Point bi-serial correlations)**			
Target-present	*r*_*pb*_ = 0.33	*r*_*pb*_ = 0.16	*r*_*pb*_ = 0.003	*r*_*pb*_ = −0.44
Target-absent	*r*_*pb*_ = 0.30	*r*_*pb*_ = 0.26	*r*_*pb*_ = 0.24	*r*_*pb*_ = 0.55

*After a Bonferroni correction was applied, a value of p < 0.006 was considered significant*.

## Discussion

Again, in contrast to previous studies (e.g., Memon and Rose, 2002; Karageorge and Zajac, 2011), we found that verbalization prior to identification had a detrimental effect on the subsequent “target-present” lineup performance in *both* children and adults. The “*content*” and “*processing shift*” accounts might both explain the presence of the VOE. However, no relationship was found between the content of the description (i.e., either quantitatively or qualitatively) and identification accuracy either in the “target-present” or in the “target-absent” lineups. Such results suggest that it is not the content of the description but, rather, the shift to featural processing caused by the verbalization that is important in order for the VOE to occur.

In addition, results of this second experiment match only partially with the “*criterion shift*” account. Indeed, the analyses performed to examine the kind of errors (i.e., misses or false alarms) occurring when the target was present in the lineup without delay (i.e., data from Experiments 1 and 2) showed that the proportion of miss responses was higher in the “description” condition than in the “control” condition and that this effect was not modulated by age. This finding is in agreement with the “*criterion shift*” account, which proposes a higher number of misses in a “target-present” lineup after verbalization because recognition criterion would become stricter. However, at variance with the “*criterion shift*” account, the prediction that false alarms would be less frequent in the “description” condition than in the “control” condition for the “target-absent” lineup was not supported by the data. Indeed, the present results indicated that false alarms occurred more (and not less) frequently in the “description” condition than in the “control” condition. Moreover, we found that the VOE (i.e., a higher level of correct performance in the “control” condition than in the “description” condition) occurred for the “target-present” lineup but also for the “target-absent” lineup both in children and adults, which again is not in line with the “*criterion shift*” account. Indeed, following this account, the recognition criterion would be expected to become stricter after verbalization, leading participants to be reluctant to choose someone in the lineup and thus to make more incorrect rejections when the target was present but more correct rejections when the target was absent. Finally, it is interesting to note that the present results suggest that children were not more susceptible than adults to choosing someone in the lineup when the target was absent, which is also in contrast with previous findings (e.g., Memon and Rose, 2002; Karageorge and Zajac, 2011).

## General discussion

Studies examining the influence of face description on subsequent identification performance in children have reported no VOE (Memon and Rose, 2002; Karageorge and Zajac, 2011). Nevertheless, it would seem premature to conclude that the VOE, described in adults so many times (see Meissner and Brigham, 2001; Meissner et al., 2008) does not affect children. Indeed, a closer examination of the research evaluating the occurrence of the VOE in children shows that these studies have contained some methodological shortcomings precluding any definitive conclusion. More precisely, in Memon and Rose's (2002) study (and similarly in Zajac and Karageorge, 2009; Karageorge and Zajac, 2011) study, which did not directly manipulate the description vs. no-description conditions but which examined the impact of spontaneously generated descriptions on identification performance), no control group of adults (in which a VOE had originally been shown) was tested. This makes it difficult to rule out the possibility that a VOE could actually have occurred in these conditions. Moreover, the procedure included a design that either contained a stimulus (e.g., the picture of a dog) that might act as a distractor or might impair the processing of the target face for some participants (i.e., a non-standardized presentation duration of the target face for all the participants), and a rather long delay (24 h) was inserted between encoding and verbalization. These procedural features could, for instance, be responsible for the production of verbal descriptions that were too short to elicit any VOE.

One aim of the present study was to re-examine the occurrence of the VOE in children through two experiments in which (1) adult groups were included as well as several groups of children, (2) an immediate test was used, (3) any strong potential distractor was avoided, (4) presentation duration of the to-be-remembered face was standardized, and (5) a large sample of participants was recruited to increase the statistical power of the study (overall, a total of 829 participants was included in the two experiments). Results from both experiments are unambiguous. A VOE occurred in the groups of children as well as in the groups of adults, and there was clearly no Age × Condition interaction. This VOE occurred for immediate recognition (Experiments 1 and 2) and for delayed recognition (Experiment 1), without Delay X Condition interaction. Finally, the VOE also occurred for “target-present” lineups (Experiments 1 and 2) and for the “target-absent” lineups (Experiment 2), without Target Presence × Condition interaction. In short, the results demonstrate that the VOE may occur in 7–8, 10–11, and 13–14-year-old children.

How can the discrepancy between the present results and those of previous studies (Memon and Rose, 2002; Zajac and Karageorge, 2009; Karageorge and Zajac, 2011) be explained? One major difference between these two previous studies and the present study that could explain the discrepancy is the nature of the encoded event. Indeed, although we used a method that better allowed us to control motivation and the opportunity to process the target face (i.e., we presented a video of a target presented in the center of the screen and no other interfering characters such as another person or a dog, for instance) instead of a staged event. The task used here may be seen as less ecologically relevant than the one used in the Memon and Rose (2002) study. However, by using such an artificial task here, it was possible to demonstrate that the VOE does exist in 7–8, 10–11, and 13–14-year-old children. This demonstration raises the question of the conditions of occurrence of the VOE in children. Further research is necessary to circumscribe better under which conditions this phenomenon may occur even in more ecologically valid situations.

The second aim of our study was to assess the competing “*content*,” “*processing shift*,” and “*criterion shift*” accounts of the VOE. Let us now examine how much the present data may help to evaluate each of these hypotheses. Two aspects of results from the current experiments do not support the “*content*” account. Firstly, according to this account, a correlation between, on the one hand, the quality or the quantity of descriptors in verbalization and, on the other hand, identification performance would be expected. No such relationship was found either in Experiment 1 or in Experiment 2 in any age group or in any delay condition. Secondly, according to the “*content*” account, when a delay is inserted between verbalization and the face recognition task, the fading memory of the content of the verbalization is less likely to have a detrimental effect on recognition performance. Again, our results were not consistent with this prediction. Indeed, no release of the VOE was found in the “*postdescription* delay” condition (see Experiment 1).

The absence of correlation between descriptor accuracy and identification performance could be consistent with the “*processing shift*” account. Indeed, according to this hypothesis, it is not the quantity or the quality of description that is important but the mere fact of producing a verbal description of a face, this production being responsible for a shift from configural to featural processing. However, like the “*content*” account, the “*processing shift*” account predicted a release of the VOE in the “*postdescription*” condition because participants would be more likely to shift back to configural processing when a long delay is inserted between description and face recognition. However, no such release occurred here. It is possible that some methodological details might explain these contrasting results. Indeed, in experiments showing a release of the VOE after “*postdescription* delay,” participants have been engaged in an unrelated cognitive task prior to identification. For instance, Finger (2002) replaced the unfilled delay condition (such as the one used in our study) with a distracting task (i.e., inviting participants either to listen to music or to work on a maze). This distracting task was probably more likely to elicit a release of retroactive interference created by the description task more likely to occur. Further research is needed in order to examine this specific issue.

Similarly, several predictions made from the “*criterion shift*” account were also not verified in the present study. Firstly, if this account is correct, verbal description would lead participants to use a stricter recognition decision, making them produce more incorrect rejections when the target is present and more correct rejections when the target is absent, compared with the control condition. Our results did not entirely fit this prediction. Indeed, on the one hand, when the target was present, the proportions of incorrect rejections were higher in the “description” condition than in the “control” condition for all groups of participants. However, on the other hand, there were fewer correct rejections in the “description” condition than in the “control” condition when the target was absent.

In conclusion, from the results of the two experiments, we were not able to definitely contrast the various accounts of the VOE, although the data seem to rather support the “*processing shift*” account. However, we did find evidence of verbal overshadowing in children and adults with “immediate” and “delayed” testing conditions or with “target-present” and “target-absent” lineups. These results clearly challenge the notion that the VOE does not occur in children.

### Conflict of interest statement

The authors declare that the research was conducted in the absence of any commercial or financial relationships that could be construed as a potential conflict of interest.

## References

[B1] Brown C. Lloyd-Jones T. J. (2005). Verbal facilitation of face recognition. Mem. Cognition 33, 1442–1456 10.3758/BF03193377 16615392

[B2] Bruck M. Ceci S. J. (1999). The suggestibility of children's memory. Annu. Rev. Psychol. 50, 419–439 10.1146/annurev.psych.50.1.419 10074684

[B3] Carey S. Diamond R. (1977). From piecemeal to configural representation of faces. Science 195, 312–318 10.1126/science.831281 831281

[B4] Carey S. Diamond R. (1994). Are faces perceived as configurations more by adults than by children? Vis. Cogn. 1, 253–274 10.1080/13506289408402302

[B5] Ceci S. J. Bruck M. (1993). The suggestibility of the child witness: a historical review and synthesis. Psychol. Bull. 113, 403–439 10.1037/0033-2909.113.3.403 8316609

[B6] Chin J. M. Schooler J. W. (2008). Why do words hurt? Content, process, and criterion shift accounts of verbal overshadowing. Eur. J. Cog. Psychol. 20, 396–413 10.1080/09541440701728623

[B7] Clare J. Lewandowsky S. (2004). Verbalizing facial memory: criterion effects in verbal overshadowing. J. Exp. Psychol. Learn. 30, 739–755 10.1037/0278-7393.30.4.739 15238020

[B8] Davies G. Flin R. (1988). The accuracy and suggestibility of child witnesses, in Issues in Criminology and Legal Psychology, eds Davies G. M. Drinkwater J. (Leicester: BPS Publications), 21–34

[B9] Davies G. Tarrant A. Flin R. (1989). Close encounters of the witness kind: children's memory for a simulated health inspection. Brit. J. Psychol. 80, 415–429 10.1111/j.2044-8295.1989.tb02333.x 2597935

[B10] Dekle D. J. Beale C. R. Elliot R. Huneycutt D (1996). Children as witnesses: a comparison of lineup versus showup identification methods. Appl. Cogn. Psych. 10, 1–12 10.1002/(SICI)1099-0720(199602)10:1<1::AID-ACP354>3.3.CO;2-P

[B11] Dent H. R. Stephenson G. M. (1979). An experimental study of the effectiveness of different techniques of questioning child witnesses. Brit. J. S. Clin. Psyc. 18, 41–51 10.1111/j.2044-8260.1979.tb00302.x 3955271

[B12] Fallshore M. Schooler J. W. (1995). Verbal vulnerability of perceptual expertise. J. Exp. Psychol. Learn. 21, 1608–1623 10.1037/0278-7393.21.6.1608 7490581

[B13] Finger K. (2002). Mazes and music: using perceptual processing to release verbal overshadowing. Appl. Cogn. Psych. 16, 887–896 10.1002/acp.922

[B14] Finger K. Pezdek K. (1999). The effect of the cognitive interview on face identification accuracy: release from verbal overshadowing. J. Appl. Psychol. 84, 340–348 10.1037/0021-9010.84.3.340 10380415

[B15] Freire A. Lee K. (2001). Face recognition in 4- to 7-year-olds: processing of configural, featural, and paraphernalia information. J. Exp. Child. Psychol. 80, 347–371 10.1006/jecp.2001.2639 11689035

[B16] Gilchrist A. McKone E. (2003). Early maturity of face processing in children: local and relational distinctiveness effects in 7-year-olds. Vis. Cogn. 10, 769–793 10.1080/13506280344000022

[B17] Hutcheson G. D. Baxter J. S. Telfer K. Warden D. (1995). Child witness statement quality: question type and error of omission. Law Hum. Behav. 19, 631–648 10.1007/BF01499378

[B18] Itier R. J. Taylor M. J. (2004). Effects of repetition and configural changes on the development of face recognition processes. Dev. Sci. 7, 469–487 10.1111/j.1467-7687.2004.00367.x 15484595

[B19] Karageorge A. Zajac R. (2011). Exploring the effects of age and delay on children's person identifications: verbal descriptions, lineup performance, and the influence of wildcards. Brit. J. Psychol. 102, 161–183 10.1348/000712610X507902 21492140

[B20] Malpass R. S. Tredoux C. G. McQuiston-Surrett D. (2009). Public policy and sequential lineups. Legal Criminol. Psychol. 14, 1–12 10.1348/135532508X384102

[B21] Marin B. V. Holmes D. L. Guth M. Kovac P. (1979). The potential of children as eyewitnesses. A comparison of children and adults on eyewitness tasks. Law Hum. Behav. 3, 295–306 10.1007/BF01039808

[B22] Meissner C. A. (2002). Applied aspects of the instructional bias effect in verbal overshadowing. Appl. Cogn. Psychol. 16, 911–928 10.1002/acp.918

[B23] Meissner C. A. Brigham J. C. (2001). A meta-analysis of the verbal overshadowing effect in face identification. Appl. Cogn. Psychol. 15, 603–616 10.1002/acp.728

[B24] Meissner C. A. Brigham J. C. Kelley C. M. (2001). The influence of retrieval processes in verbal overshadowing. Mem. Cogn. 29, 176–186 10.3758/BF03195751 11277460

[B25] Meissner C. A. Sporer S. L. Susa K. J. (2008). A theoretical review and meta-analysis of the description-identification relationship in memory for faces. Eur. J. Cog. Psychol. 2, 414–455 10.1080/09541440701728581

[B26] Memon A. Rose R. (2002). Identification abilities of children: does a verbal description hurt face recognition? Psychol. Crime Law 8, 229–242 10.1080/10683160208401817

[B27] Parker J. F. Ryan V. (1993). An attempt to reduce guessing behavior in children's and adults' eyewitness identifications. Law Hum. Behav. 17, 11–26 10.1007/BF01044534

[B28] Pozzulo J. D. Lindsay R. C. L. (1998). Identification accuracy of children versus adults: a meta-analysis. Law Hum. Behav. 22, 549–570 10.1023/A:1025739514042 9833566

[B29] Pozzulo J. D. Warren K. L. (2003). Descriptions and identifications of strangers by youth and adult eyewitnesses. J. Appl. Psychol. 88, 315–323 10.1037/0021-9010.88.2.315 12731715

[B30] Sauerland M. Holub F. E. Sporer S. L. (2008). Person descriptions and person identifications: verbal overshadowing or recognition criterion shift? Eur. J. Cogn. Psychol. 20, 497–528 10.1080/09541440701728417

[B31] Schooler J. W. (2002). Verbal overshadowing produces a transfer inappropriate processing shift. Appl. Cogn. Psych. 12, 105–125 10.1002/acp.930

[B32] Schooler J. W. Engstler-Schooler T. Y. (1990). Verbal overshadowing of visual memories: some things are better left unsaid. Cogni. Psychol. 22, 36–71 10.1016/0010-0285(90)90003-M 2295225

[B33] Schooler J. W. Fiore S. Brandimonte M. A. (1997). At a loss from words: verbal overshadowing of perceptual memories, in Psychology of Learning and Motivation, ed Medin D. L. (San Diego, CA: Academic Press), 291–340 10.1016/S0079-7421(08)60505-8

[B34] Tanaka J. W. Farah M. J. (1993). Parts and wholes in face recognition. Q. J. Exp. Psychol.-A 46, 225–245 10.1080/14640749308401045 8316637

[B35] Valentine T. (1988). Upside-down faces: a review of the effect of inversion upon face recognition. Brit. J. Psychol. 79, 471–491 10.1111/j.2044-8295.1988.tb02747.x 3061544

[B36] Wechsler D. (1997). Wechsler Adult Intelligence Scale-3rd Edition (WAIS-3®). San Antonio, TX: Harcourt Assessment

[B37] Wechsler D. (2003). Wechsler Intelligence Scale for Children-4th Edition (WISC-IV®). San Antonio, TX: Harcourt Assessment

[B38] Wells G. L. Luus E. (1990). Police lineups as experiments: social methodology as a framework for properly-conducted lineups. Pers. Soc. Psychol. B. 16, 106–117 10.1177/0146167290161008

[B39] Zajac R. Karageorge A. (2009). The wildcard: a simple technique for improving children's target-absent lineup performance. Appl. Cogn. Psychol. 23, 358–368 10.1002/acp.1511

